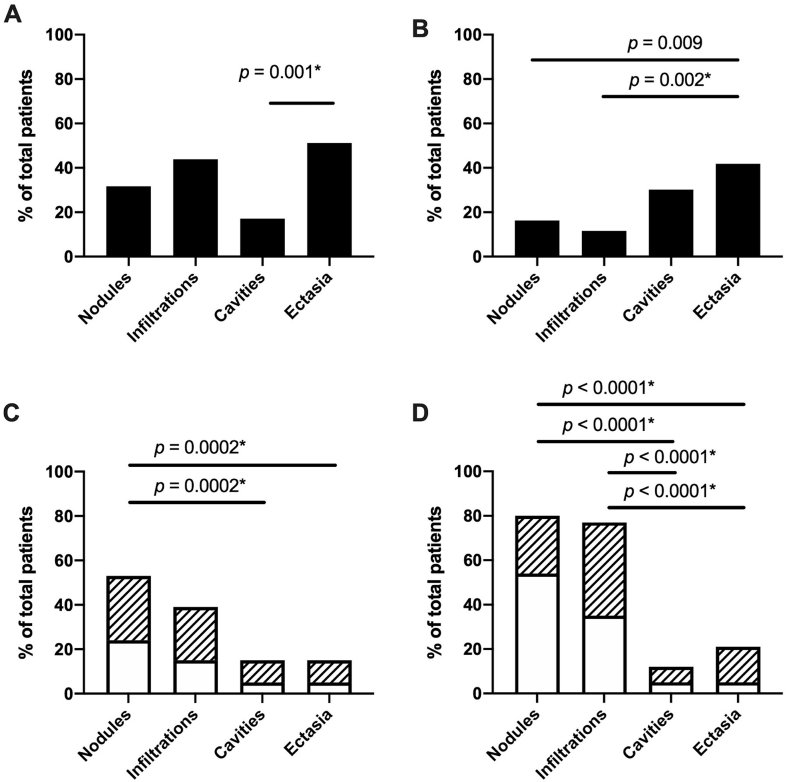# Longitudinal changes in radiographic features of pulmonary *Mycobacterium avium* complex diseases

**DOI:** 10.1016/j.heliyon.2023.e18967

**Published:** 2023-08-06

**Authors:** Chie Watanabe, Ryohei Suematsu, Tomoya Sano, Takaaki Hamamoto, Yohei Maki, Koki Ito, Hiroaki Sugiura, Hiroshi Shinmoto, Akihiko Kawana, Yoshifumi Kimizuka

**Affiliations:** a Division of Infectious Diseases and Respiratory Medicine, Department of Internal Medicine, National Defense Medical College, Saitama, Japan; b Department of Clinical Laboratory, National Defense Medical College Hospital, Saitama, Japan; c Department of Radiology, National Defense Medical College, Saitama, Japan

**Keywords:** Nontuberculous mycobacteria, Computed tomography, Mixed-effects model, Reversible lesions, Irreversible lesions

## Abstract

**Background:**

The radiographic features of *Mycobacterium avium* complex pulmonary disease (MAC-PD), a major component of nontuberculous mycobacteria, consist of a variety of lesions; however, the responsiveness of each type of radiographic factor to treatment is unclear. Thus, we evaluated the longitudinal changes of each factor in serial computed tomography (CT) images using a mixed-effects model, and investigated the radiographic transition in patients with MAC-PD whose progress could be followed.

**Methods:**

In this retrospective study, eighty-four patients diagnosed with MAC-PD and with yearly CT records were recruited after a review of 328 medical records with culture-positive MAC in respiratory specimens. The study participants were divided into two groups: treatment (n = 43) and no-treatment (n = 41) groups. Radiographic images were scored using the nodule (N), infiltration (I), cavity (C), ectasis (E) scoring system. Longitudinal changes in each radiographic lesion factor were analyzed using a mixed-effects model in treated and untreated patients.

**Results:**

All factors tended to progress without treatment, and significant longitudinal changes were observed in the N, I, and E factors (N: *p* = 0.010, I: *p* = 0.004, E: *p* < 0.001). Although treatment tended to improve N and I in radiographic images (N: *p* = 0.006, I: *p* = 0.203), cavities and ectasis progressed, regardless of treatment (C: *p* = 0.057 and E: *p* = 0.033).

**Conclusion:**

Radiographic changes of MAC-PD can be categorized into reversible (nodules and infiltrations) and irreversible (cavities and ectasis) lesions. Early treatment may prevent the accumulation of irreversible factors.

## Highlights

• *Mycobacterium avium* complex (MAC) lung changes were scored in serial CT scans.
• The nodule (N), infiltration (I), cavity (C), ectasis (E) scoring system was used.
• Nodules and infiltrations were reversible; however, ectasis was irreversible.
• Early treatment may prevent progression of irreversible factors in MAC lungs.

## 1 Background

The incidence of pulmonary nontuberculous mycobacteria (NTM) is reported to have increased recently, especially in developed countries [1,2]. In Japan, the incidence of NTM exceeded that of tuberculosis in 2014 [3]. *Mycobacterium* species distribution differs by geographic area; and *Mycobacterium avium* complex (MAC) composed of *M. avium* and *M. intracellulare,* which have similar clinical characteristics, accounts for the majority of cases of pulmonary NTM infections in countries in the Pan Pacific Region [[2], [3], [4], [5]]. Radiographic findings of nontuberculous mycobacterial pulmonary disease (MAC-PD) gradually worsen when untreated, but can improve with proper treatment [6]. Imaging tests are useful for evaluating the state of the disease. MAC-PD causes a variety of radiographic lesions [[7], [8], [9]]; however, the responsiveness of each radiographic factor to treatment is unclear. The knowledge of this responsiveness would be useful in managing the disease. Although several retrospective studies have been conducted to evaluate the pathology of MAC-PD, few longitudinal evaluations of the radiographic findings have been conducted because of the difference in follow-up period or frequency of imaging examinations due to the long disease duration. Therefore, we conducted a retrospective cohort study to evaluate the longitudinal changes of each lesion factor in serial computed tomography (CT) images using a mixed-effects model, and investigated the radiographic transition in all the patients with MAC-PD whose progress could be followed. To the best of our knowledge, this is the first study of its kind.

## 2 Methods

### 2.1 Patients

Among 328 patients at the National Defense Medical College Hospital, Japan, with culture-positive MAC from respiratory specimens submitted between January 1, 2009 and December 31, 2018, we identified 196 confirmed cases of MAC-PD diagnosed based on the criteria of ATS/ERS/ESCMID/IDSA [7]. Of the 196 patients, we included patients based on the following inclusion criteria: (a) had >12 months of follow-up period (including treatment period in the treatment group), (b) had no history of pulmonary surgery or administration of antibiotic at diagnosis, and (c) had yearly CT records from diagnosis. A total of 112 patients with factors that could affect the association between longitudinal radiographic findings and treatment responsiveness were excluded (Supplemental Fig. S1). Finally, 84 patients were included in the study. The study participants were divided into two groups: treatment (n = 43) and no-treatment (n = 41) groups. Patients who were treated with an appropriate macrolide-containing multidrug regimen were assigned to the treatment group, and the rest were assigned to the no-treatment group.

All patient data were fully anonymized before being accessed. The study protocol was reviewed and approved by the ethics committee of National Defense Medical College (No. 4037). The requirement for informed consent was waived because of the retrospective study design.

### 2.2 Radiographic evaluation

The CT interpretation was conducted by several board-certified pulmonologists with over 10 years of clinical experience using the NICE scoring system [10]. The pulmonologists were blinded to the patients’ treatment status, and supervised by board-certified radiologists. The pulmonologists evaluated both sides of the three zones of lung fields on a four-level scale (0–4) based on the percentage of the area occupied by each finding: nodules (N), infiltration (I), cavities (C), and ectasis (E). Each score was calculated by summing the results from the two reviewers randomly selected by several pulmonologists. Furthermore, as a qualitative evaluation for each lesion factor during the follow-up period, cases were classified into four categories: “improved” (only decrease in the score), “dormancy” (no change in the score), “exacerbation” (only increase in the score), and “fluctuation” (both an increase and decrease in the score).

### 2.3 Statistical analysis

The results were reported as the mean ± standard deviation (SD) for continuous variables. Continuous variables were compared using the Mann-Whitney *U* test. For the comparisons of the changes in each radiographic lesion factor, we used Fisher's exact test with the *p*-value adjusted using the Bonferroni correction for multiple comparisons. In addition, the overall trend in longitudinal radiographic changes was assessed using a mixed-effects model stratified by treatment status, statistically correcting for differences in the follow-up period of each patient. The first three analyses were performed using GraphPad Prism 8.4.3 (GraphPad Software, LLC, San Diego, CA, USA), and the mixed-effects analysis was performed using SPSS Statistics version 1.0.0.1508 (IBM Corp, Armonk, NY, USA).

## 3 Results

### 3.1 Patient characteristics at diagnosis

The study group was predominantly non-smokers, middle-aged, and females (Table 1). The NICE score on diagnosis was significantly higher in the treatment group (N: *p* < 0.001, I: *p* = 0.002) (Fig. 1A–D). The N, I, and E scores tended to be high on both sides of the middle and lower lung fields (Supplemental Figs. S2 and S3). In the treatment group, the time from diagnosis to the start of treatment was 172.0 ± 296.7 days. The total number of treatment days was 1231 ± 739.2 days, and the interval between CT scans was every 304.2 ± 74.9 days (average 1.28 scans/year).

**Table 1:** Patient characteristics (N = 84).

Characteristic	Value
Sex, n (%)	
Male	32 (38.1)
Female	52 (61.9)
Diagnostic method, n (%)	
Bronchoscopy	60 (71.4)
Sputum	24 (28.6)
Radiographic features, n (%)	
NB type	78 (92.9)
FC type	6 (7.1)
Age (years), mean ± SD	66.8 ± 10.4
Body mass index (kg/m^2^), median ± SD	19.7 ± 2.6
Smoking status Current + former smoker, n (%)	32 (38.0)
Underlying disease, n (%)	
Cancer	25 (29.8)
Tuberculosis	8 (9.5)
COPD	7 (8.3)
Diabetes mellitus	7 (8.3)
Sinusitis	6 (7.1)
Collagen disease	11 (13.1)
Use of immunosuppressant, n (%)	15 (17.9)
Blood tests	
White blood cells (/μL), mean ± SD	6092.8 ± 2181.7
Neutrophil, mean ± SD	4061.1 ± 2056.0
C reactive protein (mg/dL), mean ± SD	0.5 ± 1.7
KL-6 (IU/L), mean ± SD	300.7 ± 177.1
Alb (g/dL), mean ± SD	4.1 ± 0.4
Anti-GPL-core IgA antibody positive, n (%)	36 (64.3)
IGRA positive, n (%)	6 (12.2)
Pulmonary function	
VC (L), mean ± SD	3.4 ± 3.0
%VC (%), mean ± SD	100.7 ± 14.7
FVC (L), mean ± SD	2.8 ± 2.6
%FVC (%), mean ± SD	99.1 ± 14.2
FEV1 (L), mean ± SD	2.0 ± 0.5
%FEV1 (%), mean ± SD	101.2 ± 20.2
FEV1% (G) (%), mean ± SD	74.6 ± 10.9
Peak Flow (L/min), mean ± SD	5.7 ± 1.5
DLCO (mL/min/mmHg), mean ± SD	19.1 ± 17.7
DLCO/VA (mL/min/mmHg/L), mean ± SD	6.6 ± 12.9
Species isolated, n (%)	
*Mycobacterium avium*	66 (78.6)
*Mycobacterium intracellulare*	17 (20.2)
Unidentified species	1 (1.2)
Treatment with a macrolide-containing multidrug regimen, n (%)	43 (51.2)

Alb, albumin; COPD, chronic obstructive pulmonary disease; DLCO, diffusing capacity for carbon monoxide; FC, fibrocavitary; FEV1, forced expiratory volume in 1 s; FEV1%(G), Gänsler's FEV1% (= FEV1/VC x 100); FVC, forced vital capacity; GPL, glycopeptidolipid; IGRA, interferon-gamma release assay; KL-6, Krebs von den Lungen; NB, nodular/bronchiectasis; SD, standard deviation; VA, alveolar ventilation; VC, vital capacity.

**Fig. 1:**
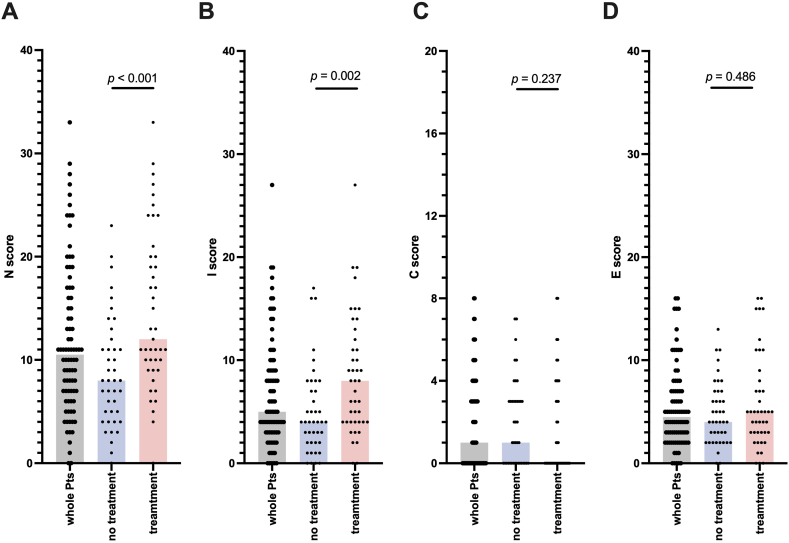
Comparison of N, I, C, and E scores at diagnosis: sum of each radiographic factor In radiographic evaluation at diagnosis using the NICE scoring system, the total scores for the entire lung field of the factors are represented (A–D). The vertical line shows the score for each factor: (N) nodule, (I) infiltration, (C) cavitation, and (E) ectasis. The gray, blue, and red bars represent all patients, no-treatment, and treatment groups, respectively. Bars with the same factors were compared using Mann-Whitney *U* test. The *p*-values for each graph are also shown.

### 3.2 Characteristics of longitudinal changes in radiographic images

#### 3.2.1 Comparison of trends in radiographic factors with and without treatment

We evaluated longitudinal changes in each factor of the NICE score using a mixed-effects model and compared between treatment and no-treatment groups (Fig. 2). In the no-treatment group, the N (Fig. 2A), I (Fig. 2B), C (Fig. 2C), and E (Fig. 2D) factors tended to worsen, and significant differences were observed in the N, I, and E factors (N: *p* = 0.010, I: *p* = 0.004, E: *p* < 0.001). In the treatment group, both the N (Fig. 2E) and I (Fig. 2F) factors improved (N: *p* = 0.006, I: *p* = 0.203). In contrast, the C (Fig. 2G) and E (Fig. 2H) factors worsened (C: *p* = 0.057 and E: *p* = 0.033). However, the change in the C factor was low, regardless of treatment administration. The difference between the treatment and no-treatment groups was significantly greater for the N (*p* < 0.001, Fig. 2A vs. 2E) and I (*p* = 0.008, Fig. 2B vs. 2F) factors, than the C (p = 0.401, Fig. 2C vs. 2G) and E (p = 0.997, Fig. 2D vs. 2H) factors, indicating that cavities and ectasis responded poorly to treatment.

**Fig. 2:**
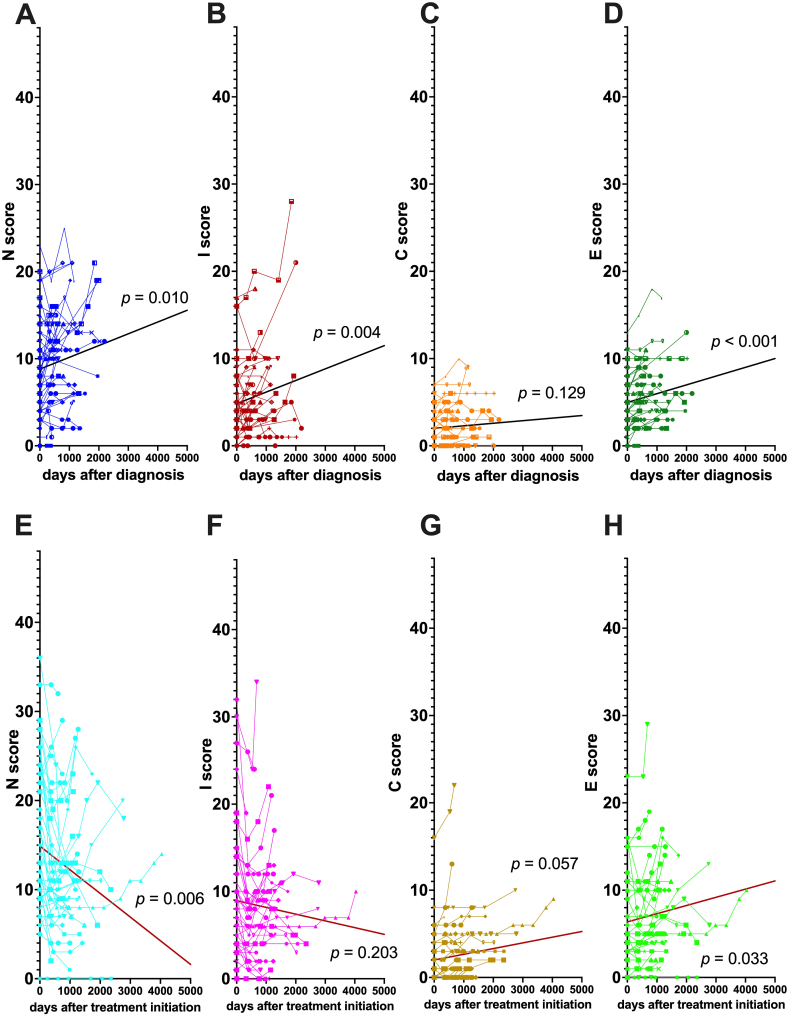
Trends of N, I, C, and E factors in treatment and no-treatment groups Graphs A, B, C, and D represent the changes in the no-treatment group of the nodules (N), infiltration (I), cavitation (C), and ectasis (E) factors, respectively. Graphs E, F, G, and H represent the changes in the N, I, C, and E factors, respectively, in the treatment group. The horizontal axis shows the time since diagnosis (A, B, C, and D) and time since treatment initiation (E, F, G, and H). The vertical axis shows the sum of scores for the N, I, C, and E factors in each patient. The bold line in each graph represents the average profile of the group using the mixed-effects model. *P* values < 0.05 indicate that the changes in the scores of the lesion factors in the group were statistically significant.

#### 3.2.2 Comparison of categorical evaluation in radiographic factors with and without treatment

Additionally, we classified cases into four categories depending on the variation in the lesion scores during the follow-up period. The changes in each lesion category of the N, I, C, and E scores are shown in the no-treatment and treatment groups (Table 2). The N and I factors had a greater tendency to fluctuate than the C and E factors. The N and I factors improved in the treatment and no-treatment groups, but the rate of improvement was higher in the treatment group. The E factor had a relatively high rate of exacerbation, regardless of treatment status. The C factor had a high rate of dormancy in both the treatment and no-treatment groups, which was similar to the trend evaluation.

**Table 2:** Trends in the NICE scores in the treatment and the no-treatment groups^a^.

	No-treatment (n = 41)								Treatment (n = 43)							
	Nodule		Infiltration		Cavity		Ectasis		Nodule		Infiltration		Cavity		Ectasis	
**Exacerbation**	14	(31.4)	18	(43.9)	7	(17.1)	21	(51.2)	9	(20.9)	7	(16.3)	13	(30.2)	15	(34.9)
**Fluctuation**	12	(29.3)	10	(24.3)	4	(9.8)	4	(9.8)	10	(23.3)	15	(34.8)	3	(7.0)	4	(9.3)
**Improvement**	10	(24.4)	6	(14.6)	2	(4.9)	2	(4.9)	22	(51.2)	16	(37.2)	2	(4.7)	7	(16.3)
**Dormancy**	5	(12.2)	7	(17.1)	28	(68.3)	14	(3.4)	2	(4.7)	5	(11.6)	25	(58.1)	17	(39.5)

a The trends in the NICE scores were classified into four categories (exacerbation, fluctuation, improvement, and dormancy) depending on the variation of each regional score during the follow-up period. Data are presented as n (%).

The rate of exacerbation was high for all lesion factors except the C score in the no-treatment group (statistically significant between C and E: *p* = 0.001) (Supplemental Fig. S4A: no-treatment group), and the exacerbation rate of the N and I factors was lower in the treatment group (statistically significant between N and E: *p* = 0.009, I and E: *p* = 0.002, respectively) (Supplemental Fig. S4B: treatment group). We also evaluated the “reversibility tendency” by adding the rate of improvement and fluctuation, which showed higher reversibility in the N and I scores than in the C and E scores, both in the no-treatment (statistically significant between N and C: *p* = 0.0002, N and E: *p* = 0.0002) (Supplemental Fig. S4C) and treatment (statistically significant between N and E: *p* < 0.0001, N and C: *p* < 0.0001, I and C: *p* < 0.0001, and I and E: *p* < 0.0001) (Supplemental Fig. S4D) groups. These results show that treatment may improve N and I scores; however, C and E scores continue to worsen even after treatment.

## 4 Discussion

In this study, we focused on the sequential changes in CT images quantitatively and qualitatively. To our knowledge, this is the first report to demonstrate the utility of a mixed-effects model showing the relationship between radiographic lesion findings and treatment status of MAC-PD patients by comparing the longitudinal changes in treated and untreated groups.

As shown in Table 1, the study group was predominantly non-smokers, middle-aged, and females, similar to previous reports [7]. The NICE score on diagnosis was significantly higher in the treatment group (Fig. 1), which was similar to our previous study [8]. The N, I, and E scores tended to be high on both sides of the middle and lower lung fields (Supplemental Figs. S2 and S3), which is consistent with the fact that radiographic findings of MAC tend to concentrate in the middle lobe and lingula, especially in the nodular/bronchiectasis type. These suggest that the radiographic characteristics led by this scoring system are consistent with the typical profile of MAC-PD [7].

All the radiographic factors evaluated by the average profiles of the no-treatment group showed significant worsening, which is consistent with our previous study which found that the radiographic findings of untreated MAC-PD patients gradually worsened [8,9]. Furthermore, although treatment improved the N and I factors, the C and E factors responded poorly to treatment. Although it has been previously reported that treatment can reduce the rate of worsening of N and I scores in mild cases [8], the reversibility of the N and I factors was not only an event in mild cases but was also observed in severe cases of this study. Moreover, there was no significant difference in the score tendencies of the C and E factors between the treatment and no-treatment groups, which suggests that the C and E factors may continue to worsen, even if treatment is given.

Additionally, we conducted a qualitative evaluation of radiographic variation in each patient which showed that the rate of exacerbation of the E factor during the follow-up periods was significantly higher than that of the other factors; whereas the reversibility of the N and I factors was higher than that of the other factors during the same period. The cavitary area (C factor) was more localized than the other lesion factors, and in many patients the score did not change during the follow-up period.

Based on these findings, the longitudinal changes in MAC-PD lesions can be hypothesized to follow a “two-track pathway” (Fig. 3). Each pulmonary lesion of MAC-PD can be categorized into two tracks: one containing N and I factors “reversible elements”, and another with E factors “irreversible elements”. Clinically, cavities are known to be formed by communication between the airways and lumen of pyogenic granulomas led by a growing nodule or as a result of progressive bronchodilation [11,12]. Therefore, the lesion factors in both tracks may progress to cavitation. Hence, it might be important to initiate treatment early to prevent the accumulation of irreversible changes that are difficult to improve.

**Fig. 3:**
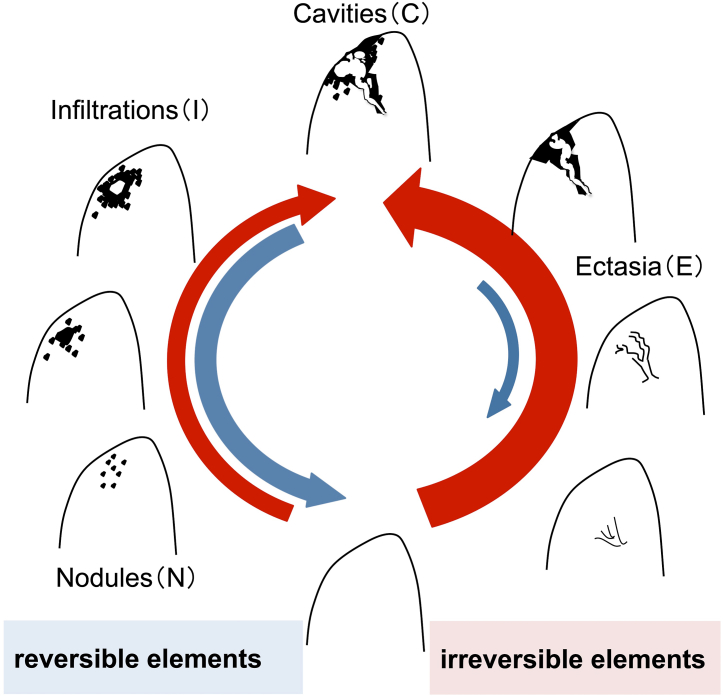
Hypothetical relationship between each radiographic factor: the “two-track pathway” We presumed that the radiographic lesion factors of MAC-PD could be categorized into two tracks: nodules (N) and infiltration (I) factors, which are reversible and indicate inflammation and granuloma formation in bronchiolitis, and ectasis (E) factors, which have a strong tendency to exacerbate. Both tracks can subsequently change into cavitation (C) factors as they progress. We hypothesized that longitudinal changes in the radiographic lesion factors on CT images of MAC-PD patients may consist of these two tracks: a reversible and an irreversible component.

Our study had some limitations. First, it was a retrospective study. There were differences in the follow-up period and interval of radiographic examinations, depending on the primary care physician. However, this point was corrected statistically as much as possible using the mixed-effects model. Second, patients may have a history of subclinical conditions; and the influence of a history of pulmonary disease, such as pulmonary tuberculosis, on the imaging score cannot be completely ruled out. Third, this study was conducted on a relatively small sample of patients attending a single facility, which means that institutional characteristics, such as actively conducting bronchoscopy and referring patients to other hospitals for follow-up after diagnosis, might have affected the study results. Based on the results of this study, further multicenter research is warranted to clarify the pathophysiology of this disease.

In conclusion, in this study, we demonstrated the usefulness of a mixed-effects model for the evaluation of the MAC-PD radiographic findings for the longitudinal quantitative change despite the varying follow-up periods among patients. This methodology could also be applied to other quantitative scoring systems, such as the Reiff score which is used to assess the severity of bronchiectasis [13]. Further, use of the mixed-effects model to evaluate the radiographic findings of MAC-PD provides useful insights into the pathophysiology of MAC-PD.

## Ethics statement

The study protocol was reviewed and approved by the ethics committee of National Defense Medical College (No. 4037).

## Author contribution statement

Chie Watanabe: Analyzed and interpreted the data; Contributed reagents, materials, analysis tools or data; Wrote the paper.

Ryohei Suematsu: Analyzed and interpreted the data; Contributed reagents, materials, analysis tools or data.

Tomoya Sano; Takaaki Hamamoto; Yohei Maki; Koki Ito; Hiroaki Sugiura; Hiroshi Shinmoto; Akihiko Kawana: Contributed reagents, materials, analysis tools or data.

Yoshifumi Kimizuka: Conceived and designed the experiments; Analyzed and interpreted the data; Contributed reagents, materials, analysis tools or data; Wrote the paper.

## Data availability statement

Data will be made available on request.

## Declaration of competing interest

The authors declare that they have no known competing financial interests or personal relationships that could have appeared to influence the work reported in this paper.

## Figures and Tables

**figs1:**
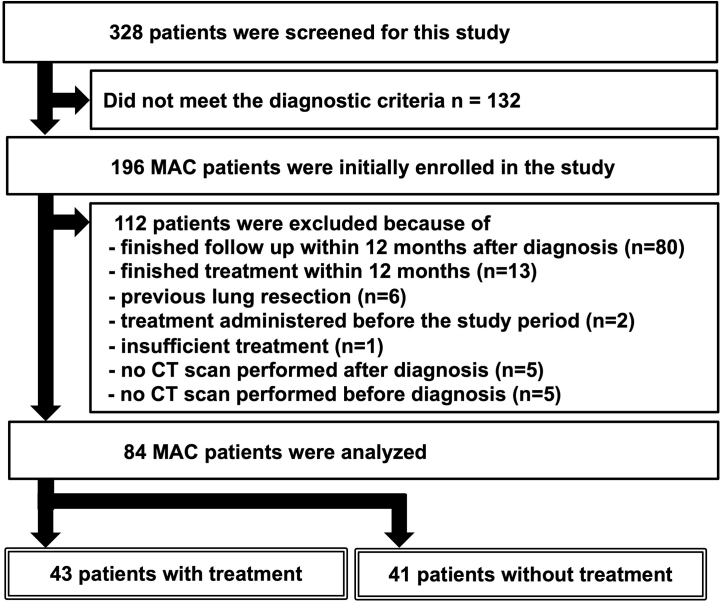


**figs2:**
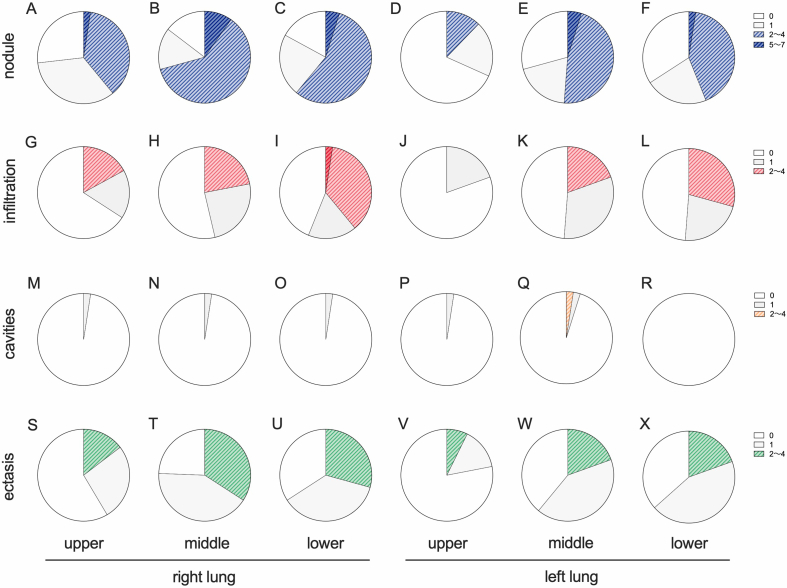


**figs3:**
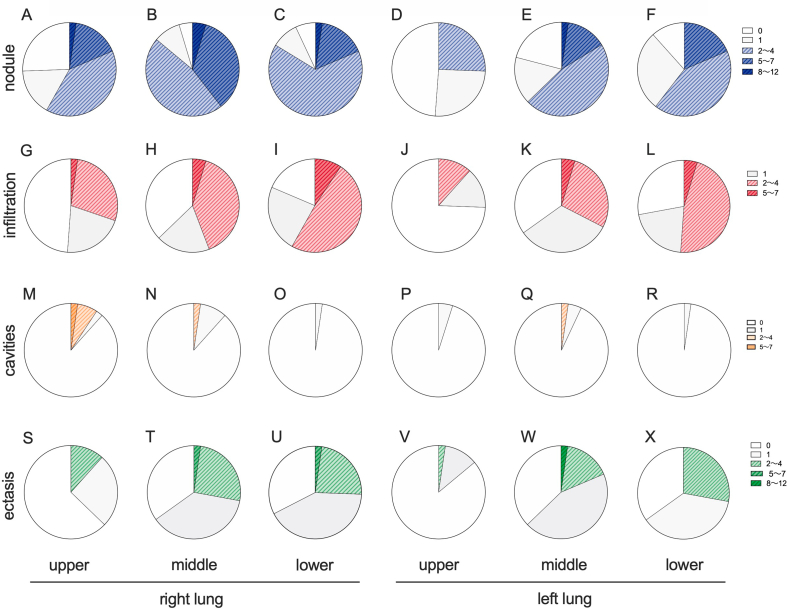


**figs4:**